# Surgical Management of Familial Trigeminal Neuralgia With Different Inheritance Patterns: A Case Report

**DOI:** 10.3389/fneur.2018.00316

**Published:** 2018-05-07

**Authors:** Claudia Cervera-Martinez, Jose J. Martinez-Manrique, Rogelio Revuelta-Gutierrez

**Affiliations:** Instituto Nacional de Neurología y Neurocirugía (INNN), Mexico City, Mexico

**Keywords:** trigeminal neuralgia, familial, microvascular decompression, arachnoid adhesions, inheritance patterns

## Abstract

**Introduction:**

Trigeminal neuralgia is a disorder characterized by unilateral electric shock-like pain, distributed in one or more trigeminal nerve branches and triggered by usually innocuous stimuli. Among the few case reports and literature reviews on familial trigeminal neuralgia (FTN), the results of several suggest the involvement of genes associated with biochemical alterations or atherosclerotic vascular malformations.

**Background:**

We present four cases of FTN within two families (family A: two brothers; family B: two sisters). All patients were submitted to surgical treatment by the same surgeon.

**Discussion:**

Cases 1 and 2 (family A) exhibited FTN with an uncommon autosomal recessive pattern and clinical features consistent with previous literature reviews and case reports. However, in cases 3 and 4 (family B), we found FTN with a dominant autosomal pattern and an unusual physiopathology characterized by arachnoid adhesions.

**Conclusion:**

We conclude, in this case report, that there are several inheritance patterns as well as physiopathology that may be involved in FTN, and that both patterns described in our reported cases were successfully managed with surgery.

## Introduction

According to the International Classification of Headache Disorders 3rd edition (ICHD-3) (1), trigeminal neuralgia (TN) is a disorder characterized by unilateral electric shock-like pain, distributed in one or more trigeminal nerve branch, triggered by normally innocuous stimuli such as chewing, shaving, or superficial touch to facial skin—even breeze contact (2, 3).

There is a gender predominance for females in TN of 1.7:1.6. The incidence of TN is 4–13 in every 100,000 people, a rate which gradually increases with advanced age (3). TN is an infrequent disorder in younger people, with those under 20 years of age accounting for only 1% of all reported cases (4, 5).

There is a subclassification for TN in the ICHD-3, which divides the disorder into three categories. The first category, classical TN, is likely caused by neurovascular compression, seen in 80–90% of cases (6). In the second category, Secondary TN is caused by an underlying disease. Finally, idiopathic TN has no known cause and presents with no reported abnormalities in magnetic resonance imaging (MRI) or electrophysiological tests (3).

Familial trigeminal neuralgia (FTN) is not officially coded in the ICHD-3 and does not fit into any subclassification. In fact, FTN has only been described in case reports since it is a very rare condition representing only 1–2% of all cases (7).

Unlike TN, FTN has a gender predominance of males at 53:47. In terms of laterality, symptoms of FTN present unilaterally in 18%, while 19% exhibit symptoms bilaterally, even though there is a higher frequency of bilateral symptom presentation in some studies. The FTN onset is usually around 43 years of age (8). FTN usually exhibits an autosomal dominant form of inheritance (9).

There are several theories about the etiology of FTN; several genes associated with an anticipation phenomenon in the early stages of life have been studied. Findings linking secondary TN with FTN in patients with Charcot–Marie–Tooth disease (9, 10) has lead Kirkpatrick and colleagues to propose that premature atherosclerotic changes in the vessels is associated with biochemical changes, such as Charcot–Marie–Tooth disease, resulting from alterations in calcium channels, as well as a possible associations with the hormone proenkephalin A, or vascular or skull base abnormalities (2, 8).

There are very few case reports on FTN. Here, we report on two families with genetic dominant autosomal and recessive autosomal transmission of TN.

## Background

We present four cases of FTN within two families (family A: two brothers; family B: two sisters). All patients were submitted to surgical treatment by the same surgeon. Written informed consent was obtained from all patients for the publication of this case report.

### Family A

#### Case 1

**Figure 1:**
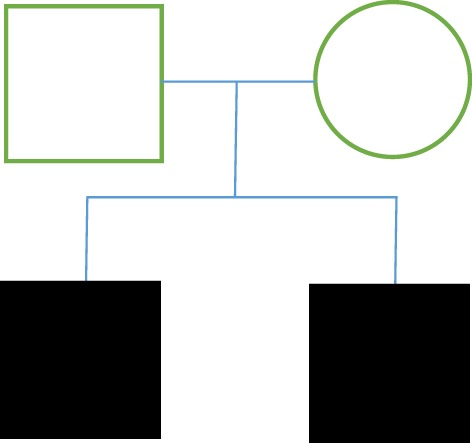
Patient pedigree: recessive autosomal pattern.

Case 1 consisted of a male patient, 71 years of age, who had developed, over 5 years, electric shock-like pain in the left mandibular that was triggered while eating or swallowing in access for 1–4 min. This patient had a family history of TN and reported having a brother with the condition. The patient’s personal pathological history consisted of hypertension for the past 30 years managed with Telmisartan (80 mg every 12 h), umbilical hernia surgery 45 years ago, and an allergy to acetylsalicylic acid. This patient was also a smoker (20 cigarettes/day) for 30 years. Clinical exam demonstrated hyperesthesia of the left maxillary and mandibular nerves (V2, V3). An MRI exam showed vascular compression of the cisternal portion of the left trigeminal nerve. The patient partially responded to carbamazepine (200 mg every 8 h); however, he still displayed outbursts of pain, and so a trigeminal nerve microvascular decompression was performed. Surgical findings: two veins that drain toward the petrous sinus. The superior cerebellar artery (SUCA) was in contact with the trigeminal nerve. Three Teflon sponges were placed between the trigeminal nerve and SUCA, and trigeminal nerve neuropraxia was performed. Following the surgery, the patient reported no further pain (Figure 1).

#### Case 2

Case 2 was a male patient, 71 years of age, with 3 years of right mandibular electric shock-like pain lasting 30 s, and triggered by chewing, eating cold foods, and shaving. This patient smoked 4 cigarettes/day for 15 years. A clinical exam of the mandibular pain demonstrated that the pain was triggered by touch in the V3. MRI results showed vascular compression of the cisternal portion of the right trigeminal nerve. This patient had been previously treated with imipramine, carbamazepine, and tramadol, with partial improvement of clinical symptoms. Trigeminal nerve microvascular decompression was performed. Surgical findings: trigeminal nerve was compressed by the SUCA and by a petrous vein branch. Teflon sponges were placed in areas of contact. Following the surgery, the patient reported no further pain (Figure 1).

### Family B

#### Case 3

In case 3, we saw a female patient, 54 years old, who reported a 20-year development of left hemifacial lancinating pain (V2, V3), of a high intensity that lasted 5–10 min, and was triggered by chewing and brushing her teeth. The patient had a family history of TN; both her father and sister reported having the condition of the clinical exam hyperesthesia in the left V2 and V3 was demonstrated. An MRI exam showed vascular compression of the cisternal portion of the left trigeminal nerve. Oral medication had no effect on the patient’s symptoms. Trigeminal nerve microvascular decompression was performed. Surgical findings: arachnoid adhesions were found at the cerebellopontine angle. We observed compression of the trigeminal nerve by the SUCA. We placed Teflon sponges in the areas of contact. Five months after the procedure, the patient presents with the same symptoms as well as left hemifacial hyperesthesia. A re-intervention was performed. Surgical findings: We found multiple arachnoid adhesions, and a misplaced Teflon sponge. The Teflon sponge is replaced. Following the surgery, the patient reports no further pain.

#### Case 4

**Figure 2:**
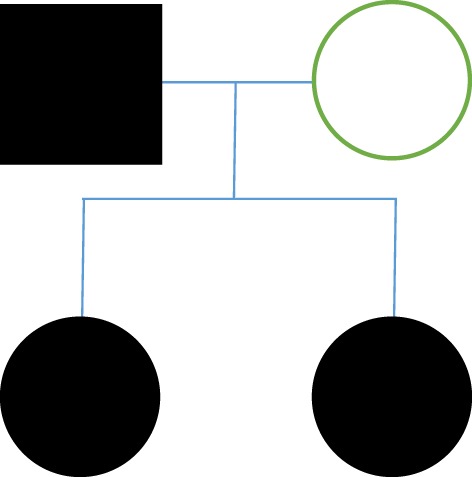
Pedigree with dominant autosomal pattern.

Case 4 consisted of a female patient of 48 years with a 15-year history of maxillary and right mandibular lancinating pain in periodic episodes. These episodes were triggered by touch, and associated with same-side epiphora and sialorrhea, and total mandibular occlusion is enabled. The patient had a family history of TN, with her father and sister having both been diagnosed with the condition. An MRI exam showed no vascular compression of the trigeminal nerves. The patient experienced partial relief of pain with medication (gabapentin and carbamazepine). We performed trigeminal nerve microvascular decompression. Surgical findings: we found multiple arachnoid adhesions at the cerebellopontine angle. The trigeminal nerve was almost completely surrounded by arachnoid adhesions. Adhesions were liberated and neuropraxia was performed; Teflon sponge fragments were placed in probable contact sites even though there was no evidence of compression. Follow surgery, the patient presented contralateral facial pain (left V1). Left microvascular decompression was performed. Surgical findings: we found multiple arachnoid adhesions at the cerebellopontine angle. The trigeminal nerve was almost completely surrounded by arachnoid adhesions. Adhesions were liberated and neuropraxia was performed; Teflon sponge fragments were placed in probable contact sites even though there was no evidence of compression (Figure 2).

## Discussion

There are few case reports about FTN in the literature, and very few case reviews. However, in this study, we describe two genetic FTN inheritance patterns in four separate cases of FTN with autosomal dominant form of inheritance being the predominant form. In family A (cases 1 and 2), both siblings are male and have a delayed onset of the disease (around their early 70s). Case 1 had a history of hypertension and atherosclerosis, and case 2 had a history of smoking. In both cases, the surgeries revealed vascular compression by the SUCA. Symptoms were completely eliminated after microvascular decompression. The results of the surgery support previous literature, which report an influence of atherosclerotic changes and associated biochemical changes on TN, which could have particular bearing on case 1. On the other hand, the inheritance pattern reported in cases 1 and 2 was a recessive autosomal inheritance pattern, which is not the pattern that has been suggested in the few FTN case reports.

In family B (cases 3 and 4), we report a different presentation of FTN than in family A. First, both patients were female with an earlier onset of the disease (late 40s and early 50s). In case 3, MRI results revealed a compression of the SUCA, and through surgery we also found multiple adhesions. There was a recurrence of symptoms 5 months after the surgical decompression, and so we performed a re-intervention. The re-intervention revealed arachnoid adhesions and a Teflon sponge displacement. Adhesions were liberated and three Teflon sponges were placed in three different contact sites. Following re-intervention surgeries, the patient reported no further symptoms.

Case 4 presented no findings of vascular compression, but several arachnoid adhesions were observed. Adhesions were successfully liberated with no further symptoms reported immediately after surgery; however, the patient developed contralateral V1 pain. In response, we performed a second contralateral microvascular decompression. Again, we found arachnoid adhesions, which were successfully liberated, and the patient reported no further symptoms. This case is classified as secondary TN, according to the ICHD-3, since there was no evidence of vascular compression.

Family B presented with a dominant autosomal pattern of inheritance with arachnoid adhesions as the apparent cause of pain. An anticipation phenomenon was also identified in this family. Based on the findings in previous literature, all characteristics of FTN in family B were uncommon, except for the inheritance pattern, which is the most commonly reported pattern in cases of FTN. The fact that there was a bilateral TN in case 4 is notable given that it has been described with only 19% frequency in FTN.

## Concluding Remarks

Familial trigeminal neuralgia is an uncommon pathology with very few reported cases. All four cases in this report were successfully submitted to surgical treatment by the same surgeon after initial unsuccessful pharmaceutical treatment. There was one surgical re-intervention required. We found two different inheritance patterns and two different physiopathologies in these four cases of FTN, and both surgical findings were successfully treated. However, it is of vital importance to continue investigating and analyzing FTN to better understand the uncommon presentation of neuralgia, and to obtain more knowledge to generate a more accurate and improved evaluation and treatment of the disease.

## Author Contributions

RR-G is the tutor of this project. CC-M was in charge of the project analysis. JM-M was in charge of data recollection.

## Conflict of Interest Statement

The authors declare that the research was conducted in the absence of any commercial or financial relationships that could be construed as a potential conflict of interest.
